# On the similarities and differences of non-traumatic sound exposure during the critical period and in adulthood

**DOI:** 10.3389/fnsys.2013.00012

**Published:** 2013-05-06

**Authors:** Jos J. Eggermont

**Affiliations:** Department of Physiology and Pharmacology, Department of Psychology, University of CalgaryCalgary, AB, Canada

**Keywords:** auditory cortex, tonotopic map, plasticity, animal, human

## Abstract

There is an almost dogmatic view of the different effects of moderate-level sound stimulation in neonatal vs. adult animals. It is often stated that exposure in neonates results in an expansion of the cortical area that responds to the frequencies present in the sound, being either pure tones or frequency modulated sounds. In contrast, recent findings on stimulating adult animals for a sufficiently long time with similar sounds show a contraction of the cortical region responding to those sounds. In this review I will suggest that most neonatal animal results have been wrongly interpreted (albeit generally not by the original authors) and that the changes caused in the critical period (CP) and in adulthood are very similar. Thus, the mechanisms leading to the cortical map changes appear to be similar in the CP and in adulthood. Despite this similarity, the changes induced in the CP are occurring faster and are generally permanent (unless extensive training paradigms to revert the changes are involved), whereas in adults the induction is slower and a slow recovery (months) to pre-exposure conditions takes place.

## Introduction

The still prevailing dogma on use-dependent adult cortical plasticity is expressed in the following (Keuroghlian and Knudsen, 2007, pp. 113–114; references removed from citation, italics are mine):

“To induce adaptive plasticity in the adult central auditory system, acoustic stimuli must be behaviorally relevant. Frequency tuning is the response property that has been used most often to document plasticity in adults. The plasticity of frequency tuning has been studied in a variety of species and with a variety of training paradigms. Most of these studies have focused on the auditory cortex, specifically the primary auditory cortex (A1), but some have studied the central nucleus of the inferior colliculus (ICC) and the medial geniculate nucleus (MGN). *The results from all of these studies agree that merely exposing adult animals to an environment dominated by a particular frequency has no effect on the representation of that frequency*. Instead, in order to alter frequency tuning in adults (without lesioning the cochlea), either the animal must be conditioned to attend to the frequency, usually accomplished through positive or negative reinforcement, or the frequency must be paired with electrical microstimulation of the brain applied directly to the circuit or to sources of modulatory input such as cholinergic and dopaminergic systems.”

It is the purpose of this review to demonstrate that the italicized statement cannot be taken at face value, and that the effect of passive sound exposure in adults surprisingly can produce changes in auditory cortex that are similar to those in critical period (CP) animals. Yet, differences remain particularly in the time it takes to induce the changes and in the potential for spontaneous recovery from the induced changes.

## Adult animal plasticity

**Figure 1:**
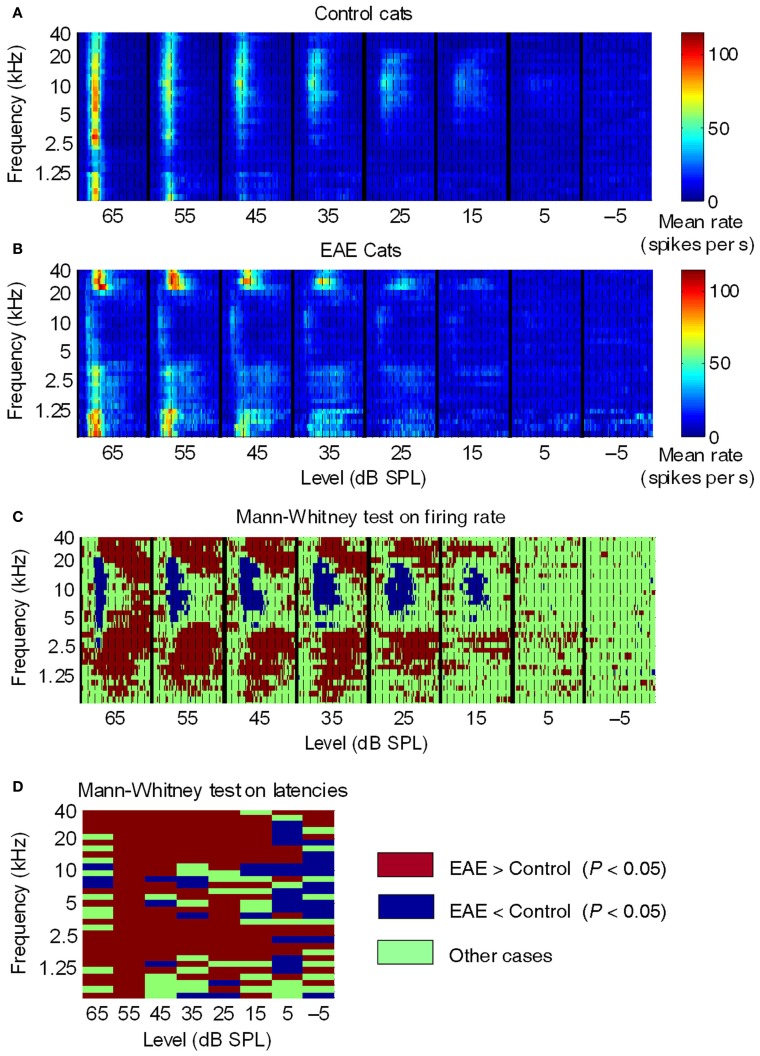
**Averaged post-stimulus time histograms across all control and EAE cats. (A,B)** Averaged PSTHs (2-ms time bins) as a function of SPL, over a 100-ms time window, in control, and EAE cats. Dashed lines, 10-ms intervals. Colored bars, mean firing rate. In control cats **(A)**, the mean response suggested that the highest sensitivity (lowest thresholds) was to frequencies around 10 kHz and that the largest responses were to frequencies between 2.5 kHz and 10 kHz. In EAE cats **(B)**, the most sensitive frequencies were those below 1.25 kHz and above 20 kHz. Note that neural responses in EAE cats were much more spread out over time compared to those in control cats. **(C,D)** Graphical representation of significant differences (Mann-Whitney test) between EAE and control groups in terms of **(C)** mean firing rate per frequency-latency bin and **(D)** mean latency per frequency-intensity bin. From Noreña et al. (2006).

In 2006 we reported on an experiment that exposed adult cats to an enhanced acoustic environment (EAE), a 4–20 kHz random multi-tone pip stimulus ensemble presented at 80 dB peSPL for ~5 months continuously (Noreña et al., 2006). The dBA equivalent level was slightly lower at 78 dB, and could thus be considered safe for long-term exposure. This was confirmed by the normal auditory brainstem response (ABR) thresholds obtained at the end of the exposure period. To our surprise we found that the neurons in A1 had ceased to respond to the majority of tone pips with frequencies between 4 and 20 kHz, the exception being a narrow band around 10 kHz. This could thus be interpreted as a contraction of the representation of the 4–20 kHz range. This finding is illustrated in Figure 1.

The figure compiles post-stimulus-time histograms (PSTHs) over 0–100 ms, and at stimulus levels of −5 to 65 dB SPL. The PSTHs are arranged according to SPL so as to form a compound response area of AI. The color scale represents mean peak firing rates in 2 ms bins. Figure 1A shows the average data for 15 control cats, and indicates that the most sensitive frequency in cat AI is around 10 kHz, which fits for frequencies over 5 kHz with data for auditory nerve fibers (Liberman, 1978) and behavior (Fay, 1988). The relatively high thresholds (relative to the cited sources) for frequencies below 5 kHz are the result of a recording bias; lower frequency neurons can be hidden in the posterior ectosylvian sulcus, i.e., unreachable with our multi-electrode arrays (two rows of four electrodes with 0.5 mm between electrodes). Because of the averaging used in these graphs, the thresholds are elevated. It is assumed that this recording bias is the same for EAE cats as well.

What can be seen in Figure 1B, showing the average PSTHs for four EAE cats, are the following dramatic points: (1) the boundary of reduced neural activity is very sharp and coincides closely with the sharp boundary of the EAE. (2) Some neural activity is remaining around 10 kHz with nearly normal thresholds. (3) For frequencies above and below the EAE range, thresholds are reduced by up to 20 dB, and peak amplitudes are strongly enhanced, compared to controls. (4) The transient response type found in the control cats is now replaced by a response lasting as long as the tone pips. This happened in the low frequency range particularly below 1.5 kHz and thus more than an octave below the low-frequency boundary of the EAE. On the high-frequency side the enhancement borders the EAE cut-off frequency. Figures 1C,D for control cats indicates that at high sound levels a broad (in frequency), short latency and short duration response occurs, and is followed by a profound post-activation suppression. The latter is at least partly the result of feed-forward inhibition via an interneuron activated by the thalamo-cortical afferents. For EAE cats the PSTHs showed long latency and sustained responses at both the high-frequency and low-frequency border of the EAE frequency range. The longer latency of these responses caused by horizontal fiber activation can be explained by the slow conduction velocity of these horizontal fibers (<0.5 m/s). The absence of post-activation suppression is a strong indicator of horizontal fiber input, as these fibers typically do not produce feed-forward inhibition (Noreña et al., 2006).

**Figure 2:**
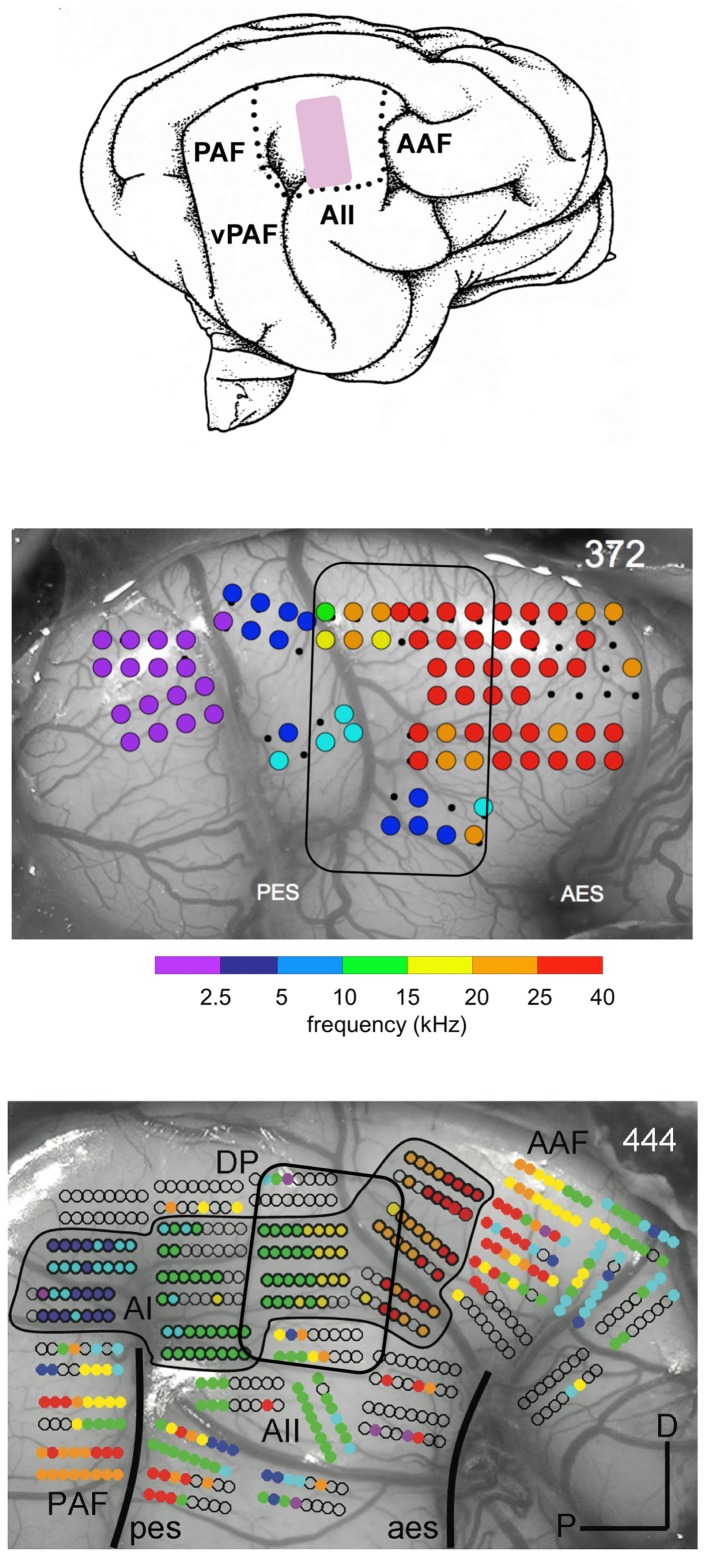
**Map of best frequencies at 65 dB SPL onto the cortical surface in a normal hearing adult cat reared in an enhanced acoustic environment (4–20 kHz) presented for at least 5 mo at 80 dB SPL (middle panel).** For comparison the map in an unexposed normal hearing cat is shown in the bottom panel. The cartoon of the auditory fields (top panel) indicates the region (pink) where the 4–20 kHz are normally represented. In the outlined region in the EAE cat there is a lack of responses to frequencies in the range of 5–20 kHz and an overrepresentation of frequencies above 25 kHz. From Eggermont (2008) and Pienkowski and Eggermont (2012).

We also found that the tonotopic map was profoundly changed by the EAE exposure (Figure 2). In the outlined region in the middle panel (same location as the pink area in the top panel) one observes a lack of responses to frequencies in the range of 5–20 kHz and an overrepresentation of higher frequencies, particularly those with CFs >25 kHz. In comparison for a normal tonotopic map (bottom panel) one can see a more gradual change from low frequency to high frequency sites. Note also the outlined region for the corresponding part of the top panel cartoon. We argued that the reorganization of the tonotopic map could be due to weakened thalamo-cortical and strengthened horizontal cortico-cortical fiber synapses onto the pyramidal cells.

**Figure 3:**
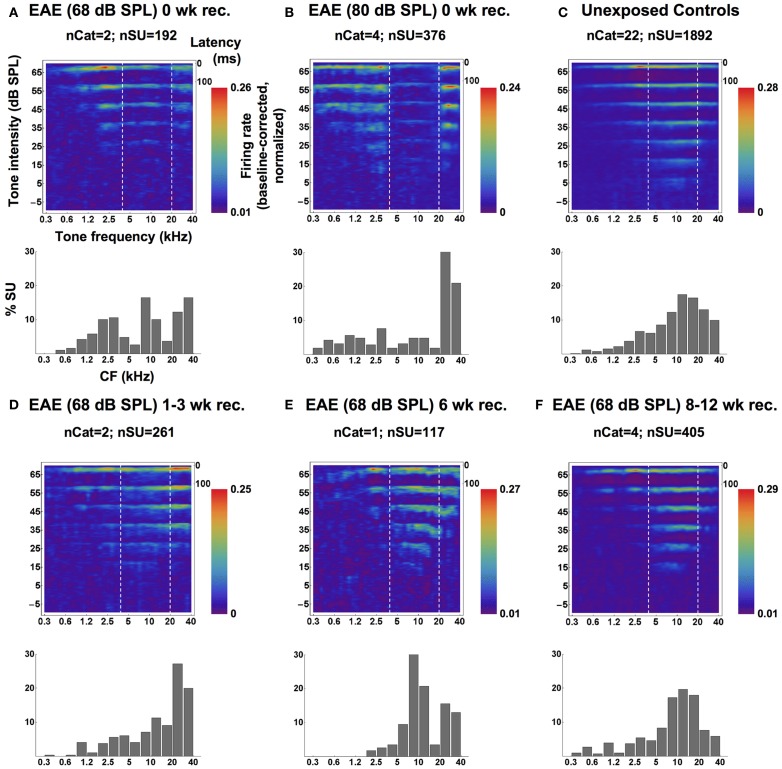
**Averaged SU-derived frequency tuning curves (top panels) and CF distributions (**bottom panels**; bin-width = 1/2 octave), measured from groups of cats immediately following the 6-weeks EAE exposure at 68 dB SPL **(A)** and a 20-weeks exposure 80 dB SPL **(B)**, from a group of unexposed controls **(C)**, and from groups of cats exposed at 68 dB SPL and allowed 1–3 weeks **(D)**, 6 weeks **(E)** or 8–12 weeks **(F)** of recovery.** In the top panels, averaged SU responses to individually-presented tone pips (at one of eight SPLs) are shown as smoothed PSTHs up to latencies of 100 ms (time scale in the top-right corner). Each SU response was baseline-corrected and normalized before averaging. High firing rates are represented by the red end of the color spectrum and low firing rates by the blue end, and each plot is scaled to its own extremes. Dashed white lines mark the bandwidth of the EAE. Reprinted from Pienkowski and Eggermont (2009), with permission from Elsevier.

Are these changes in responsiveness and tonotopic map the result of the very long exposure times (>5 mo) and 80 dB SPL that may be potentially damaging to the inner hair cell ribbon synapses (Kujawa and Liberman, 2009) despite normal ABR thresholds? A very recent study by Maison et al. (2013) exposed animals to a band-pass noise at 84 dB SPL for one week and found that despite no OHC loss, normal DPOAEs, and normal ABR thresholds, there was a small but significant reduction in the ABR wave I amplitude and a corresponding small reduction in ribbons per IHC, and IHC synapse survival. Our ABR data at that point were only used to estimate threshold, so we could not evaluate that. Being aware of these potential effects we then started using exposure levels well below the effective quiet level (Ward et al., 1976); “Effective quiet, the highest SPL of a noise that will neither produce a significant temporary threshold shift nor retard recovery from a TTS produced by a prior exposure to a higher level, is shown to be about 76 dB for octave bands of noise centered at 250 and 500 Hz, and around 68 dB for those centered at 1000, 2000, or 4000 Hz.” We decided to repeat these exposure experiments at more modest levels (68 dB peSPL) and shorter exposure times (6 weeks), and also checked if the response changes were genuine plastic and could spontaneously return to normal (Pienkowski and Eggermont, 2009). Again, in these experiments we did find that ABR thresholds and amplitudes at 70 dB SPL were normal. Thus, although there was a quantitative difference, we concluded that ribbon synapse loss for the 80 dB peSPL exposure was unlikely to be the cause of the findings. The compound tuning results, again in the form of PSTHs, are shown in Figure 3.

The top row of Figure 3 shows the compound responses (now rotated 90° compared to Figure 1) for **(C)** control, **(B)** EAE = 80 dB SPL, and **(A)** for 68 dB SPL. Although the suppression effect is less for the lower exposure level and duration, one can clearly see from the compound response areas and the histograms, showing the % CFs of neurons, below each panel that there are dips in the responsiveness just below 20 kHz and above 4 kHz. The percentage of neurons tuned to the 10 kHz region remained unchanged compared to control. Panels **D–E** in the bottom half of the figure show the effects of recovery in quiet after 6 weeks exposure to the EAE. They indicate that the histograms showing the percentage of neuron's CFs obtain close to normal shape only after 8–12 weeks (Figure 3F).

**Figure 4:**
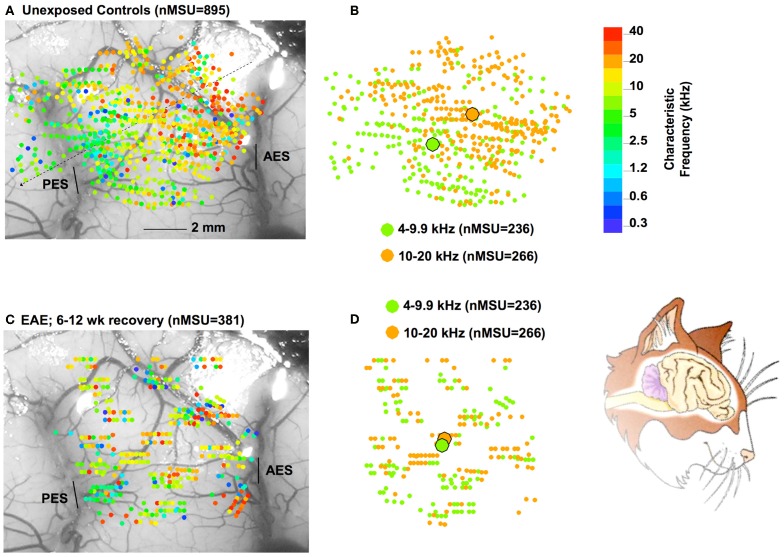
**Part **(A)** show a composite of all (*N* = 895) recording sites in 15 control cats, color-coded with respect to CF (color bar on the right).** The CFs in the range of 4–20 kHz are extracted and replotted on the right, now with only two colors, one representing the 4–9.9 kHz range and the other the 10–20 kHz range. **(B)** One observes a clear separation of the centers of gravity (large filled circles) for these two frequency regions. In part **(C)** results are plotted for 381 recording sites for cats exposed to 68 dB SPL for 6 weeks followed by 6–12 weeks of recovery in quiet. **(D)** As the data for the 4–9.9 and 10–20 kHz show, the centers of gravity now overlap, indicating a distorted tonotopic map. The cartoon on the bottom right is similarly oriented as panels **(A)** and **(C)**.

However, the cortical region affected by the EAE exposure is still not normal after 3 months of recovery in quiet, because the tonotopic map remained distorted in the 4–20 kHz region (Figure 4). In this Figure, panel **A** shows the CF of single units measured in 15 control cats with respect to recording sites in AI, plotted on one particular cat brain. One observes the gradual increase in CFs from left (caudal) to right (frontal, see inset of a cartoon of the cat's brain) as indicated by the dotted line. In panel **B**, the sites with CFs between 4 and 20 kHz are replotted and now color-coded for the lower (4–9.9 kHz region; green), and for the 10–20 kHz region (in orange). The two centers of gravity are indicated by the large filled circles. These are located quite some distance apart indicating the significant segregation of these two frequency regions. After 6–12 weeks of recovery from the EAE (panels **C**, **D**) the two frequency regions covering the EAE are overlapping as illustrated by nearly the same position of their centers of gravity. It is presently not clear if the map will return to normal after a longer waiting time, or if this would only happen after further rearing in a different acoustic environment, potentially accompanied by training (e.g., as in Zhou and Merzenich, 2007 for CP exposed animals).

**Figure 5:**
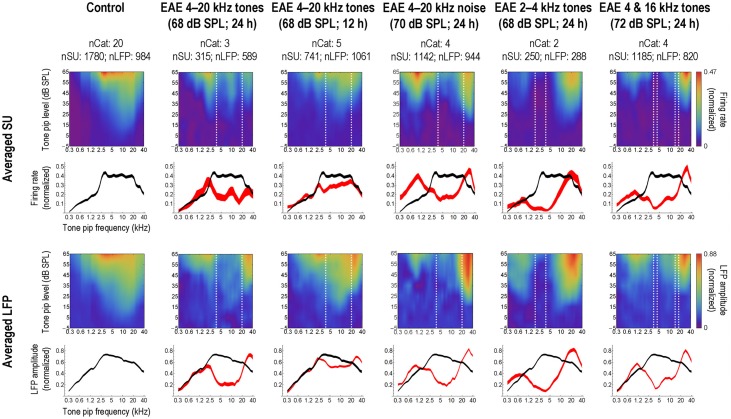
**Long-term, passive exposure of adult cats to moderate-level, bandlimited sounds decreases AI activity in the exposure frequency range.** Left-most column: population-averaged single-unit (SU; top rows) and local field potential (LFP; bottom rows) responses to tone pips, obtained from a uniform sampling of AI in a group of normal-hearing, unexposed control cats. Each SU and LFP response was normalized on its baseline-corrected maximum value prior to averaging. In the first and third rows, responses are shown as a function of tone pip frequency and intensity; strong responses are represented by the red end of the color spectrum and weak responses by the blue end (scale bars at far right apply to the entire row of plots). The second and fourth rows show averaged frequency tuning curves taken at the best SPL of each individual response; curve thickness illustrates the Bonferroni-corrected 95% confidence interval about the mean. Subsequent columns: averaged responses from groups of cats measured immediately following long-term (>5 weeks), passive exposure to various experimental acoustic environments (EAEs), as specified in the column headings. Dashed white lines on the frequency-intensity plots mark the bandwidth of the EAE. Averaged frequency tuning curves from the exposed cats are shown in red, and are compared to the control curve, again reproduced in black; where the difference between the two means is larger than the sum of their 95% confidence levels (i.e., no overlap between curves), that difference is significant at *P* < 0.05. Reprinted from Pienkowski and Eggermont (2011), with permission from Elsevier.

Thus, qualitatively, the data for 6 weeks exposure to a 68 dB SPL EAE, conform to those for the 5 months exposure to the same EAE presented at 80 dB SPL The tonotopic maps contract initially and whereas the CF-distribution of single units recovers to normal after 6 weeks in quiet, abnormalities in the tonotopic map persist. We subsequently tested several different EAEs; random multi-tone pip stimulus ensembles of (1) one-octave wide (2–4 kHz), and (2) two 1/3rd octave bands centered around 4 and 16 kHz (Pienkowski and Eggermont, 2010b), and (3) a 4–20 kHz filtered noise (Pienkowski et al., 2011) and found basically the same results (Figure 5, top two rows). In addition, the short-latency part of the averaged local field potentials (LFP) representing the thalamo-cortical input to the AI show basically the same effect (Figure 5, bottom two rows). A comparison between the 4–20 kHz multi-tone EAE and the 4–20 kHz filtered noise EAE shows some differences in the response enhancements above and below the EAE frequency range, but otherwise produced the same results. An interesting effect was noted for the EAE consisting of two 1/3rd octave bands of multi-tone frequencies; here the region with decreased spike and LFP activity was nearly the same as for the contiguous 4–20 kHz EAE.

We (Pienkowski and Eggermont, 2010a) found qualitatively similar effects of passive exposure occurred when the EAE presentation was limited to 12 h/day (Figure 5, third column). Compared to continuous exposure at the same SPL (Figure 5, second column) and over a similar duration (6–12 weeks), this intermittent exposure produced a smaller decrease in AI spike and LFP activity in response to sound frequencies in the exposure range, and an increase in LFP amplitude only for frequencies above the exposure range. As expected at these moderate exposure levels, cortical changes occurred in the absence of concomitant hearing loss (i.e., ABR threshold shifts). Since there is some overlap in the amount of change in neural activity between the intermittently exposed group and the continuously exposed group, it is expected that recovery from the effects of the intermittent exposure would also take a long time.

Recently, we addressed the use of low aggregate tone-pip presentation rates (Pienkowski et al., 2013). In that paper we stated we exposed cats to a pair of 1/3rd octave bands but with the presentation rate reduced to 2.5 pips/s in the 4 kHz band. Finding a similar suppression profile in AI with this lower density EAE, we then reduced the rate in the 16 kHz 1/3rd octave band to just 0.5 pips/s, which again failed to affect the suppression profile. Thus, exposure with stimuli containing as few as 0.5 pips/s produced or at least maintained a degree of plasticity similar to that previously observed with much denser stimuli.

Zheng (2012) exposed 50 days-old, i.e., adult, rats to a 60–70 dB SPL, 4–45 kHz noise continuously for 30 days. The tonotopic map underwent a dramatic reorganization, i.e., the systematic change from low to high CF from caudal to rostral disappeared. Behavioral testing showed that fine pitch discrimination was impaired, whereas coarse-pitch discrimination remained. Interestingly, the noise-exposed rats performed similarly in a quiet and noisy acoustic testing environment, whereas control rats performed much more poorly in background noise. This suggests that noise-exposed adult animals have adapted to perception in a noise living environment, potentially by reorganizing their tonotopic maps, and frequency tuning properties.

Also extending our studies on passive sound exposure driven plasticity in adult AI, Zhou and Merzenich (2012) exposed 3-months-old, i.e., adult, rats to pulsed noise bursts delivered at 65 dB SPL for a 2-months period. This modulated broad-spectrum noise exposure was intended to model the noise environments encountered in the industrial workplace and other modern acoustic settings. Frequency tuning curve bandwidths were generally increased in pulsed noise-exposed (PNE) rats, but changes in tonotopic maps were not reported. Significant behavioral impairments and negative cortical changes in temporal and spectral sound processing were induced in these PNE adult rats. They first examined the behavioral performance of PNE versus age-matched control rats by using temporal rate discrimination tasks. The results showed that a 2-months-long exposure to moderate-level structured noises significantly degraded these adult animals' abilities to discriminate between sound stimulus rates. These post-exposure effects persisted for at least 6 weeks after the end of noise exposure. Statistical analysis showed no significant ABR-threshold differences between PNE and control rats at any frequency determined. Response thresholds and latencies recorded at cortical sites in PNE rats did not differ from those recorded in control rats. Note the strong similarity with our earlier data. Qualitatively similar post-exposure effects were also documented even when exposure was limited to 10 h/day (as in Pienkowski and Eggermont, 2010a), an exposure regimen that better models a noisy-work/quiet-living environment. This study thus provides evidence that chronic exposure to moderate level of structured noises during adulthood can significantly and persistently impair central auditory processing and auditory-related perceptual abilities.

## Animal critical period plasticity

de Villers-Sidani et al. (2008) exposed CP rats to a 5–20 kHz band-pass noise; similar bandwidth, but a different carrier as used by 2006 Noreña et al. (2006) in adult cats, and also found a compression of the 5–20 kHz frequency range in A1. So for this stimulus there appeared to be no difference in the effect of stimulation between critical-period rats and adult cats, as we later confirmed similar results for a 4–20 kHz multi-tone stimulus and a 4–20 kHz band-pass filtered noise (Pienkowski et al., 2011). These corresponding findings prompted a new look at critical-period studies with respect to the effects of non-traumatic sound exposure, tones or noise, on tonotopic map representation. I will use a chronological approach.

### Tonal environments

Stanton and Harrison (1996) stimulated newborn kittens for 3 months using an 8 kHz (±1 kHz) FM stimulation at a level between 55 and 75 dB SPL. The exposure produced no hearing loss as was determined from ABR recordings and cortical response thresholds in adulthood (>1 year old). At this time the cortical (AI) tonotopic maps were determined and compared with those in age-matched non-exposed controls. They found a significant expansion of the 6–12 kHz region. However, this has been interpreted often as an expansion of the area of stimulation. Since this was between 7 kHz and 9 kHz, the observed expansion range was much larger. Scrutinizing their Figure 1 and comparing the unexposed control CFs recorded in the 6–12 kHz range across the three animals suggests that the expansion affected predominantly units with CF >9 kHz, i.e., the region above the stimulation frequencies. This is similar to what we found a large enhancement in responsiveness in adults (cf. Figure 1).

**Figure 6:**
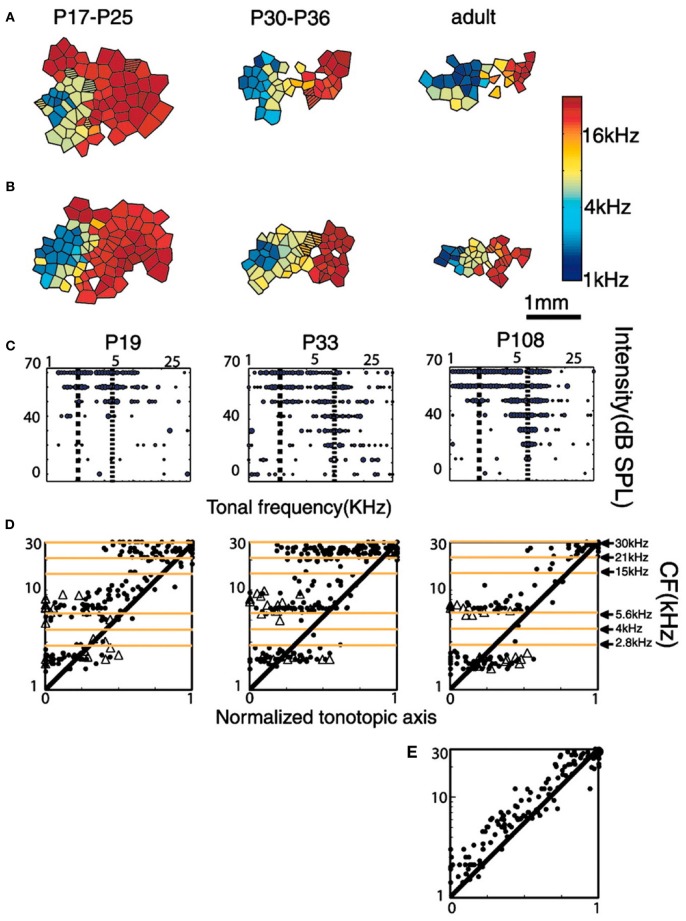
**Tonotopic organization of A1 of rats exposed to a tone sequence with two specific spectro-temporal patterns at different post-natal ages. (A,B)** Examples are auditory maps from two different rat litters reared in the critical period (P9–P30) in the presence of these sequences of sound stimuli. Post-natal ages of rats are indicated at the top of each map. **(C)** Representative tonal receptive fields obtained from A1 at different ages. Dotted lines indicate the positions of peaks within the receptive field. **(D)** Distribution of CFs (represented by solid dots) and frequencies of secondary peaks (ρ) along the tonotopic axis of the auditory cortex at different developmental stages. Note the regions of tonal stimulation indicated at the right-most graph. Also note that especially for the low-frequency tones (2.8, 4, and 5.6 kHz) the responses between 2.8 and 5.6 kHz are suppressed and those below 2.8 kHz and above 5.6 kHz are enhanced. **(E)** Distribution along the tonotopic axis of the auditory cortex in control naive adult rats (P100). Reprinted from Nakahara et al. (2004), with permission from National Academy of Sciences, USA.

Zhang et al. (2001) stimulated rats during the CP for 10–16 h/day with 25-ms tones (4 kHz or 19 kHz) at 60–70 dB SPL and at six pulses per s with 1-s intervals to minimize adaptation effects. In rats that were exposed to a pulsed 4-kHz tone, a low frequency (2–6 kHz) tuned sector emerged as early as post-natal day (P) 14, whereas, in naive rats, low-frequency representations did not appear until P18-P20. Thus, tonal stimulation did speed up maturation. At P22, the posterior zone of the exposed rat's cortex (the presumptive A1 precursor) was dominated by neurons responding selectively to frequencies clustered around 4 kHz. Another three litters of rat pups were exposed to 19-kHz pulsed tonal stimuli over the same time epoch, and with the same experimental schedule. Compared to non-exposed rats, this exposure resulted in a significant increase in the area of the posterior region in which neurons were sharply tuned to CFs centered at or near 19 kHz. The changes induced in the CP persisted into adulthood. This was later (Keuroghlian and Knudsen, 2007) interpreted as “the AI came to over-represent the experienced frequency and, in this sense became customized to the acoustic environment experienced by the individual during this sensitive period.” From close studying Zhang et al. (2001)'s Figure 6, one cannot escape the impression that the expansion related to 4 kHz stimulation is dominated by CFs from 2.6–9 kHz, whereas that to 19 kHz stimulation covers a wide range with CFs from 9–30 kHz. I interpret this as an expansion that is not at the stimulation frequency but in wide regions surrounding these frequencies.

A subsequent study from the Merzenich group (Nakahara et al., 2004) exposed rat pups through a period extending from P9 (hearing in rats starts to be functional at P12) to P30 (when the CP is presumed to be ending) to a tone sequence with two specific spectro-temporal patterns. This stimulation consisted of two sets of tone sequences with distinct temporal orders: a set of pulsed low-frequency tones presented in the order 2.5, 5.6, and 4 kHz; followed after a brief pause and a larger sound frequency jump by a set of pulsed high-frequency tones presented in the order 15, 21, and 30 kHz. Each tone lasted 30 ms with an intensity of 65 dB SPL. Interestingly, and in agreement with the non-selective expansion in rats exposed to isolated single tones (Zhang et al., 2001), the expanded representations in adulthood for low frequency stimulation here were not centered at 2.8 kHz, 4 kHz, and 5.6 kHz, but just below 2.8 kHz and just above 5.6 kHz (Figure 6). Again, results can be explained as expansions occurring above the stimulated frequencies with a contraction for the stimulated frequencies (low frequency region) or only a contraction (for high frequency stimulation).

de Villers-Sidani et al. (2007) on the other hand found for pure tone stimulation with a 7 kHz tone presented at 70 dB SPL, that the cortical region corresponding to 7 kHz ± 0.3 octave was expanded by about 20% for exposure from P11–P13 and mapping at P60. The expansion of A1 activation at 65 dB SPL ranged from 3.5–14 kHz, i.e., 1 octave on both sides of the 7 kHz tone frequency, again not limited to the exposure frequency.

Thus, with this one exception, potentially related to the particular time slot of exposure, the general finding in CP animals can be interpreted as an expansion for units with CFs above and below the stimulated frequency region, combined with a potential reduction in the cortical representation for the frequencies of stimulation.

### Noise environments

Zhang et al. (2002) exposed rat pups to pulsed (65 ms duration, once per s) broad-band noise at 65 dB SPL during P9–P28, which resulted in broader-than-normal tuning curves, in multipeaked tuning curves, and in a discontinuous tonotopic map in A1. In addition, weaker than normal temporal correlations between the discharges of nearby A1 neurons were recorded in exposed rats. In contrast, pulsed-noise exposure of rats older than P30 did not cause significant changes in auditory cortical maps. Zhou and Merzenich (2012) corroborated this by showing that exposure of 60 days old rats with pulsed noise did not affect the tonotopic map but still introduced profound behavioral changes (see above). Thus, synchronous activation of multiple frequencies appears to play a crucial role in shaping neuronal processing in the A1 during a CP. One would have expected that these synchronous activations by the noise pulses would result in synchronous firing under spontaneous conditions, however, this did not happen. This may have been a result of the discontinuity of responses within the receptive fields, albeit that the bandwidths of tuning curves at 20 dB above the threshold at CF were significantly larger than control rats.

Chang and Merzenich (2003) found that “[…] rearing infant rat pups in continuous, moderate-level noise delayed the emergence of adult-like topographic representational order and the refinement of response selectivity in the A1 long beyond normal developmental benchmarks. When those noise-reared adult rats were subsequently exposed to a pulsed pure-tone stimulus, A1 rapidly reorganized, demonstrating that exposure-driven plasticity characteristic of the CP was still ongoing”. de Villers-Sidani et al. (2008) showed that exposure with a 5–20 kHz band-pass noise in critical-period rats delayed the closure of the CP for this particular frequency range, whereas other frequency ranges all showed signs of critical-period closure.

Ranasinghe et al. (2012) tested whether exposure to pulsed noise or speech sounds in P9–P38 rats would alter neural representations and behavioral discrimination of speech. Both groups of rats were trained to discriminate speech sounds from P50–P100, and anesthetized neural responses were recorded from A1. Pulsed noise changed the frequency representation in A1 such that the cortical area was extended for frequencies below 3 kHz and reduced for frequencies above 10 kHz and increased frequency-tuning bandwidth. Speech-rearing only reduced the frequency representation for frequencies above 10 kHz to some extent. The representation of speech in A1 and behavioral discrimination of speech was little affected after pulsed-noise exposure. Exposure to passive speech during early development did not change speech sound processing either. Speech training increased A1 neuronal firing rate for speech stimuli in naïve rats, but did not increase responses in rats that experienced early exposure to pulsed-noise or speech. This suggests that speech sound processing is resistant to changes in cortical frequency tuning and tonotopic maps caused by manipulating the early acoustic environment.

The spectral, temporal, and intensive selectivity of neurons in the adult A1 is easily degraded in early post-natal life by raising rat pups in the presence of pulsed noise (Zhang et al., 2002). The non-selective frequency tuning recorded in these rats substantially endures into adulthood. By using a modified go/no-go training strategy, structured noise-reared rats were trained to identify target auditory stimuli of specific frequency from a set of distractors varying in frequency (Zhou and Merzenich, 2007). Tonotopicity and frequency-response selectivity returned to normal after this perceptual training. Changes induced by training were retained for at least 2 months after the end of training.

## Summary of animal data

Both adult and CP animals show plastic changes in auditory cortex following passive exposure to tonal or noise stimuli. The CP, in general, is considered a time period when the best neural representation of the environment is selected from among the many competing inputs that affect the maturing nervous system. The growth and function of lateral inhibitory circuits may be important for terminating the CP. The difficulty of this problem is highlighted by the fact that the closure of the early CP may be dependent on the input received (Zhang et al., 2001; Chang and Merzenich, 2003). Moreover, specific types of auditory experience can result in the CP remaining open in some parts of A1, but being closed in others (de Villers-Sidani et al., 2008), further emphasizing the fact that CPs are controlled by sensory inputs. Note that Zheng (2012) in adult rats exposed to continuous noise found a complete disappearance of tonotopic order, i.e., as if the rats had reentered a condition similar to the critical-period rats. Pulsed noise stimulation in neonatal animals disrupts the tonotopic map and broadens frequency tuning, whereas in adult animals map changes do not occur but behavioral effects related to broader frequency tuning are evident. Tonal stimulation in CP animals either expands the region of single frequency stimulation and up to an octave wide region on either side, or contacts the region of multi-tone stimulation and expands the surrounding frequencies. In adult animals, the stimulated region contracts regardless if stimulated with band-pass tonal or noise stimuli, whereas the bordering regions dramatically expand. These changes in adults spontaneously recover, those in CP animals only in the case of continuous noise, which delayed closure of the CP. For the pulsed noise or tonal stimulation in CP animals spontaneous recovery does not occur. The relationship between map changes in A1 and behavior remains unclear.

Are the EAEs really “passive” for the animals? Although they did not have to make responses they may have started listening outside the stimulation band in order to better communicate or listen to other environmental sounds. This “attention change” might have affected the responses in auditory cortex. Albeit not extensively discussed in this review, but represented in Figure 5, in our EAE series that started with the 4–20 kHz exposures (both multi-frequency tone pip stimuli or noise) we also included a 2–4 kHz multi-tone stimulus that overlapped with the dominant vocalization formants of the cats, and a combination of two 1/3rd octave bands (centered at 4 and 16 kHz) that would minimally interfere with either hearing their own vocalizations or other important environmental sounds. Yet, as Figure 5 showed, all of these EAEs produced strong suppression/enhancement effects. Furthermore, there was no enhancement for units with CFs between the two 1/3rd octave bands, as would be expected if the cats were listening outside the stimulated 4 and 16 kHz regions. This formed the basis for us to consider the exposures “passive.”

A unifying mechanism would be that stimulation suppresses neural activity at the specific frequency (ies) of stimulation, and likely by loss of lateral inhibition enhanced activity up to one octave above or below that frequency (Pienkowski and Eggermont, 2012). The exception of the expanded tonotopic map at exactly the stimulus frequency by de Villers-Sidani et al. (2007) could imply that cortical lateral inhibition is not fully formed at this early age.

## Relevance for humans

### Human neonates

It is not exactly known whether there are similar CP s in human auditory development, but from the cochlear implant (CI) literature one may derive CPs for the necessity of auditory stimulation for binaural hearing [<2 years of unilateral hearing (i.e., one CI; Gordon et al., 2012)]; for the development of certain auditory evoked response components (i.e., N1; >3 years of deafness under the age of six; Ponton and Eggermont, 2001), and for normal language development (Svirsky et al., 2000). Conductive hearing loss in children is a major determinant of language delay and may potentially cause long-lasting deficits.

The human cochlea is fully developed by 24 weeks of gestation. A blink startle response can first be elicited (acoustically) at 24–25 weeks and is constantly present at 28 weeks. Hearing thresholds are 40 dB SPL at 27–28 weeks and reach the adult threshold of 13.5 dB SPL by 42 weeks of gestation (Birnholz and Benacerrah, 1983). Early born preterm children often end up in the neonatal intensive care unit (NICU), and quite often they show signs of auditory neuropathy and sensorineural hearing loss; however, even in case they do not, they may have other neurological problems from which they only very slowly recover (Marlow et al., 2005).

A busy NICU is by default a noisy environment. Noise is also present in the confines of an isolette or incubator. A big issue is the so far largely unknown effect of prolonged noise exposure in the NICU on the neonatal brain. Whereas it has been established that this does not cause hearing loss, it may still have profound effects on hearing, as the animal studies suggest (Zhang et al., 2001; Chang and Merzenich, 2003). In neonatal and adult animals, band-pass noise exposure leads to contracting tonotopic maps surrounded by expanding tonotopic maps (Pienkowski and Eggermont, 2012). Potential extrapolations can be drawn that pertain to human auditory development. Several studies of long-term outcomes in NICU graduates cite speech and language problems (Stjernqvist and Svenningsen, 1999; Marlow et al., 2005; Kern and Gayraud, 2007). However, few studies have specifically linked them with noise type and levels.

### Human adults

Would the adult auditory cortical plasticity induced by the noise- and tone-EAEs in animals also develop in humans exposed to moderately loud environments in the real world? Although our 4–20 kHz noise and tone stimuli have near-identical long-term power spectra, they sound different, as the tone ensemble has a much more variable short-term frequency spectrum and a low-pass modulation spectrum. Continuous exposure to either stimulus produced a comparable suppression of neural activity in AI, suggesting that mixes of tonal and noise sounds (i.e., a more realistic, real-world noise) could have similar effects. There are several caveats, however. All of our stimuli were sharply band-limited, whereas the power spectra of natural sounds would fall off more gradually; thus, the edge effect that was proposed to enhance suppression should be smaller for more realistic sounds. This was recently confirmed for both factory noise and multi-tone EAEs with only 12 dB/oct slopes (Pienkowski et al., 2013). Another potential factor was that our exposures were less structured (more random) than typical sources of real-world noise, and may thus have been easier to “habituate to” (Kjellberg, 1990). Perhaps the most important factor would be the duration of the exposure. As mentioned above, a decrease in the suppression effect was found when the exposure was reduced from 24 to 12 h/day; a further decrease might be expected from 12 to 8 h or less. A similar reduction in the amount of suppression was found after exposure to EAEs with 12 dB/oct slopes compared to those with very steep slopes. The very long recovery times will however still result in a demonstrable effect after several weeks of exposure. The reduced effect may, furthermore, be more than offset by an intermittent, real-world recreational noise exposure that occurs over years or decades, rather than weeks or months as in our laboratory. If so, would the time course of the reversal of plasticity also be more protracted than that observed in our studies? Would full reversal even be possible, given that longer-term exposure led to a complete reorganization of the tonotopic map in AI (Noreña et al., 2006)? This awaits further investigation.

Kujala et al. (2004) reported that long-term exposure to noise had a persistent effect on central auditory processing that underlies behavioral deficits. They found that speech and sound discrimination was impaired in noise-exposed individuals, as indicated by behavioral responses and the auditory mismatch negativity (MMN) brain response. These subjects were healthy individuals exposed to occupational noise for several years, with peripheral hearing (i.e., audiological status) that did not, however, differ from that of individuals in the control group not exposed to long-term noise. These results demonstrated that long-term exposure to noise had long-lasting detrimental effects on central auditory processing and attention control. They recorded auditory evoked potentials from 10 healthy noise-exposed workers (exposure duration >5 years) and 10 matched controls with 32-channel EEG in two conditions, one including standard and deviant speech sounds, the other non-speech sounds, with novel sounds in both. The MMN was larger to non-speech than speech sounds in control subjects, while it did not differ between the sound types in the noise-exposed subjects. Thus, subpathological changes in cortical responses to sounds may occur even in subjects without a peripheral damage but continuously exposed to noisy auditory environments. Furthermore, long-term exposure to noise had a persistent effect on the brain organization of speech processing and attention control (Kujala and Brattico, 2009). These results indicate the need to re-evaluate which noise levels can be considered safe for brain functions and raise concerns on the speech and cognitive abilities of individuals living in noisy environments.

These combined animal and human studies thus demonstrated that several aspects of mature AI function remain impaired over the long-term by an uninterrupted passive exposure to a moderate-level, spectrally-EAE. These results combined also argue strongly for the importance of more completely defining these potential hazards of moderate-level noise exposure that cannot be detected with the standard audiogram. This could have serious implications for persistently noisy work/living places, even at levels considerably below those that presently are required by law to use sound protection.

### Conflict of interest statement

The author declares that the research was conducted in the absence of any commercial or financial relationships that could be construed as a potential conflict of interest.
